# Notification that new names of prokaryotes, new combinations and new taxonomic opinions have appeared in volume 74, part 9 of the IJSEM

**DOI:** 10.1099/ijsem.0.006556

**Published:** 2024-12-17

**Authors:** Aharon Oren, Markus Göker

**Affiliations:** 1 The Institute of Life Sciences, The Hebrew University of Jerusalem, The Edmond J. Safra Campus, 9190401 Jerusalem, Israel; 2 Leibniz Institute DSMZ – German Collection of Microorganisms and Cell Cultures, Inhoffenstrasse 7B, 38124 Braunschweig, Germany

This listing of names of prokaryotes, published in a previous issue of the *IJSEM*, is provided as a service to microbiologists to assist in the recognition of new names and combinations. This procedure was proposed by the Judicial Commission [1]. The names given herein are listed according to the rules of priority (i.e. the time and date of publication of the articles on the journal’s platform and the order of valid publication of names in the original articles) [2].

**Table:** 

Order of article publication	Name/authors	Proposed as	Article no.	Page no.
1	*Eshraghiella* Fatahi-Bafghi 2024	gen. nov.	006509	5
1	*Eshraghiella crossota* (Moore *et al*. 1976) Fatahi-Bafghi 2024	comb. nov. [basonym: *Butyrivibrio crossotus* Moore *et al*. 1976 (Approved Lists 1980)]	006509	7
2	*Aequorivita flava* Chen *et al*. 2024	sp. nov.	006513	9
3	*Hyphobacterium marinum* Zhao *et al*. 2024	sp. nov.	006512	8
3	*Hyphobacterium lacteum* Zhao *et al*. 2024	sp. nov.	006512	8
4	*Rhizobium aouanii* Hsouna *et al*. 2024	sp. nov.	006515	10
5	*Luteirhabdus salina* (Chen *et al*. 2023) Huang and Lai 2024	comb. nov. (basonym: *Thermobacterium salinum* Chen *et al*. 2023)	006519	4
6	*Paenimyroides marinum* (Song *et al*. 2013) Li 2024	comb. nov. (basonym: *Flavobacterium marinum* Song *et al*. 2013)^*^	006522	1
7	*Nocardiopsis codii* Girão *et al*. 2024	sp. nov.	006483	12
7	*Rhodococcus chondri* Girão *et al*. 2024	sp. nov.	006483	12
8	*Amycolatopsis nalaikhensis* Oyuntsetseg and Kim 2024	sp. nov.	006511	9
8	*Amycolatopsis carbonis* Oyuntsetseg and Kim 2024	sp. nov.	006511	9
9	*Luteipulveratus flavus* Xiong *et al*. 2024	sp. nov.	006518	6
10	*Polaribacter ponticola* Baek *et al*. 2024	sp. nov.	006526	7
11	*Streptomyces castrisilvae* Widén *et al*. 2024	sp. nov.	006514	12
11	*Streptomyces glycanivorans* Widén *et al*. 2024	sp. nov.	006514	12
12	*Lactobacillus amylovorus* subsp. *amylovorus* (Nakamura 1981) Yamane *et al*. 2024	comb. nov. (basonym: *Lactobacillus amylovorus* Nakamura 1981)	006517	8
12	*Lactobacillus amylovorus* subsp*. animalis* Yamane *et al*. 2024^†^	subsp. nov.	006517	8
13	*Pseudohoeflea coraliihabitans* Yu *et al*. 2024	sp. nov.	006453	6
14	*Haloarcula saliterrae* (Straková *et al*. 2024) Straková *et al*. 2024	comb. nov. (basonym: *Halomicroarcula saliterrae* Straková *et al*. 2024)	006510	6
14	*Haloarcula onubensis* (Straková *et al*. 2024) Straková *et al*. 2024	comb. nov. (basonym: *Halomicroarcula onubensis* Straková *et al*. 2024)	006510	7
15	*Anoxybacteroides* Patel *et al*. 2024^‡^	gen. nov.	006528	15
15	*Anoxybacteroides amylolyticum* corrig. (Poli *et al*. 2006) Patel *et al*. 2024^§^	comb. nov. (basonym: *Anoxybacillus amylolyticus* Poli *et al*. 2006)	006528	15
15	*Anoxybacteroides contaminans* (De Clerck *et al*. 2004) Patel *et al*. 2024	comb. nov. (basonym: *Anoxybacillus contaminans* De Clerck *et al*. 2004)	006528	15
15	*Anoxybacteroides geothermale* corrig. (Filippidou *et al*. 2016) Patel *et al*. 2024^|^	comb. nov. (basonym: *Anoxybacillus geothermalis* Filippidou *et al*. 2016)	006528	15
15	*Anoxybacteroides rupiense* corrig. (Derekova *et al*. 2008) Patel *et al*. 2024^¶^	comb. nov. (basonym: *Anoxybacillus rupiensis* Derekova *et al*. 2008)	006528	15
15	*Anoxybacteroides tepidamans* (Schäffer *et al*. 2004) Patel *et al*. 2024	comb. nov. (basonym: *Geobacillus tepidamans* Schäffer *et al*. 2004)	006528	16
15	*Anoxybacteroides voinovskiense* corrig. (Yumoto *et al*. 2004) Patel *et al*. 2024^#^	comb. nov. (basonym: *Anoxybacillus voinovskiensis* Yumoto *et al*. 2004)	006528	16
15	*Paranoxybacillus* Patel *et al*. 2024	gen. nov.	006528	16
15	*Paranoxybacillus vitaminiphilus* (Zhang *et al*. 2013) Patel *et al*. 2024^**^	comb. nov. (basonym: *Anoxybacillus vitaminiphilus* Zhang *et al*. 2013)	006528	16
15	*Thermaerobacillus* Patel *et al*. 2024	gen. nov.	006528	16
15	*Thermaerobacillus caldiproteolyticus* (Coorevits *et al*. 2012) Patel *et al*. 2024	comb. nov. (basonym: *Anoxybacillus caldiproteolyticus* Coorevits *et al*. 2012)	006528	17
15	*Brevibacillus sedimenti* Patel *et al*. 2024	nom. nov. (basonym: *Anoxybacillus sediminis* Khan *et al*. 2019)	006528	17
16	*Xanthomonas cerealis* Tambong *et al*. 2024	sp. nov.	006523	8
16	*Xanthomonas graminis* Tambong *et al*. 2024	sp. nov.	006523	9
17	*Dongia sedimenti* Jiang *et al*. 2024	sp. nov.	006532	5
18	*Virgibacillus tibetensis* Li *et al*. 2024	sp. nov.	006525	7
19	*Microbulbifer spongiae* Ishaq *et al*. 2024	sp. nov.	006521	10
20	*Paralimibaculum* Kawano *et al*. 2024	gen. nov.	006530	10
20	*Paralimibaculum aggregatum* Kawano *et al*. 2024	sp. nov.	006530	10
20	*Biformimicrobium* Kawano *et al*. 2024	gen. nov.	006530	11
20	*Biformimicrobium ophioploci* Kawano *et al*. 2024	sp. nov.	006530	11
21	*Utexia* Phillips *et al*. 2024	gen. nov.	006516	13
21	*Utexia brackfieldae* Phillips *et al*. 2024	sp. nov.	006516	13
21	*Orbus sturtevantii* Phillips *et al*. 2024	sp. nov.	006516	14
21	*Orbus wheelerorum* Phillips *et al*. 2024	sp. nov.	006516	14
21	*Orbus mooreae* Phillips *et al*. 2024	sp. nov.	006516	14
22	*Nitrosotalea* Lehtovirta-Morley *et al*. 2024^**^	gen. nov.	006387	7
22	*Nitrosotalea devaniterrae* Lehtovirta-Morley *et al*. 2024^**^	sp. nov.	006387	7
22	*Nitrosotalea sinensis* Lehtovirta-Morley *et al*. 2024^**^	sp. nov.	006387	7
22	*Nitrosotaleaceae* Lehtovirta-Morley *et al*. 2024^**^	fam. nov.	006387	7
22	*Nitrosotaleales* Lehtovirta-Morley *et al*. 2024^**^	ord. nov.	006387	7

* Earlier legitimate homotypic synonyms: *Flavobacterium marinum* Song *et al.* 2013 and *Myroides aquimaris* García-López *et al.* 2020. Earlier illegitimate homotypic synonym: *Paenimyroides aquimaris* (García-López *et al.* 2020) Zhang *et al.* 2023.

† The name is illegitimate as it contravenes Rule 12b of the ICNP.

‡ The etymology must give N.L. neut. n. instead of N.L. masc. n. [3]

§ The List Editors have corrected *amylolyticus* to *amylolyticum* (a.my.lo.ly’ti.cum. Gr. neut. n. *amylon*, starch; Gr. masc. adj. *lytikos*, able to dissolve; N.L. neut. adj. *amylolyticum*, starch dissolving).

| The List Editors have corrected *geothermalis* to *geothermale* (ge.o.ther.ma’le. Gr. fem. n. *ge*, earth; N.L. fem. adj. *thermalis*, of thermal properties or origin; N.L. neut. adj. *geothermale*, from hot earth, from a geothermal site).

¶ The List Editors have corrected *rupiensis* to *rupiense* (ru.pi.en’se. N.L. neut. adj. *rupiense*, originating from Rupi Basin, referring to the place of isolation of the type strain).

# The List Editors have corrected *voinovskiensis* to *voinovskiense* (voi.nov.ki.en’se. N.L. neut. adj. *voinovkiense*, from Voinovskie, referring to the Voinovskie Hot Springs, the place of isolation).

** Previously described as a *Candidatus* taxon.

The following taxonomic opinions (i.e. the emendation of circumscriptions or the creation of synonyms) do not affect the status of a name under the International Code of Nomenclature of Prokaryotes. These taxonomic opinions can neither be considered approved nor disapproved by the International Committee on Systematics of Prokaryotes.

**Table:** 

Name/authors:	Article no.	Page no.
*Anoxybacillaceae* Chuvochina *et al*. 2024 emend. Patel *et al*. 2024	006528	14
*Anoxybacillus* Pikuta *et al*. 2000 emend. Patel *et al*. 2024	006528	14
*Hyphobacterium* Sun *et al*. 2017 emend. Zhao *et al*. 2014	006512	7
*Lactobacillus amylovorus* Nakamura 1981 emend. Yanana *et al*. 2024	006517	8
*Polaribacter sejongensis* Kim *et al*. 2013 emend. Baek *et al*. 2024	006526	8
*Polaribacter undariae* Park *et al*. 2015 pro synon. *Polaribacter sejongensis* Kim *et al*. 2013	006526	7
*Pseudohoeflea* Hyeon *et al*. 2017 emend. Yu *et al*. 2014	006453	6
*Parageobacillus caldoxylosilyticus* (Ahmad *et al*. 2000) Aliyu *et al*. 2019 pro synon. *Saccharococcus thermophilus* Nystrand 1984	006528	17
*Saccharococcus caldoxylosilyticus* Ahmad *et al*. 2000 pro synon. *Saccharococcus thermophilus* Nystrand 1984	006528	17
